# Effect of timing on baseline quality of life scores among surgical cancer patients

**DOI:** 10.1186/s13104-018-3312-y

**Published:** 2018-04-02

**Authors:** Daniel Steffens, Michael Solomon, Kenneth Vuong, Lyndal Alchin, Rachael Roberts, Cherry Koh, Jane Young

**Affiliations:** 1 Surgical Outcomes Research Centre (SOuRCe), Royal Prince Alfred Hospital (RPAH), PO Box M157, Missenden Road, Sydney, NSW 2050 Australia; 2 Sydney Medical School, The University of Sydney, Sydney, Australia; 3 Institute of Academic Surgery (IAS), Royal Prince Alfred Hospital, Sydney, Australia

**Keywords:** Quality of life, Preoperative, Postoperative, Surgery, SF-36, Cancer

## Abstract

**Objectives:**

To investigate differences between quality of life (QoL) scores obtained preoperatively or recalled in the early postoperative period amongst patients undergoing major cancer surgery.

**Results:**

Of the 283 patients included, 133 completed their baseline QoL questionnaire preoperatively and 150 postoperatively. Patient groups were broadly comparable in terms of age however the preoperative group had a lower proportion of patients from non-English speaking backgrounds. There were important and statistically significant differences between mean scores for physical health (overall physical health, physical functioning and role physical domains) and mental health (overall mental health and mental health domains) between pre- and postoperative groups. There were no differences for other domain-specific scores (bodily pain, general health, vitality, social functioning and role emotional).

## Introduction

Improvements in patient selection, surgical skills, technology, and multidisciplinary care have led to dramatic improvements in survival rates for patients undergoing major cancer surgery [1]. For this reason, more patients will live with permanent consequences of the disease and treatment, including pain, bowel and sexual dysfunction, psychological distress, faecal, urinary incontinence and body issues; resulting in drastic changes in their lifestyle and impacting their health-related quality of life (QoL) [2, 3].

Longitudinal studies that assess patient-reported outcomes such as QoL provide valuable information about the impact of treatment-related consequences on patients’ daily life. In such longitudinal research, baseline pre-intervention assessment of QoL provides the reference level from which improvements or deterioration can be assessed. Additionally, preoperative QoL is considered a prognostic factor for many conditions, and has been shown to correlate with surgical outcomes and long-term survival [4].

In some circumstances however, it is not always possible to collect baseline data preoperatively for surgical patients, particularly those admitted as emergency cases. For elective admissions, the trend towards patient admission on the day of surgery leaves little time for QoL assessment prior to the procedure. This combined with multiple medical, surgical, nursing and allied health assessments make the time for QoL assessments limited. In longitudinal studies that investigate changes in QoL over time, statistical imputation methods can be used for missing follow-up data [5]. However, it is extremely difficult to accurately impute missing baseline data, which could potentially weaken the validity of the results and conclusions [6].

An alternative and pragmatic approach to obtain ‘missed’ preoperative QoL information is to ask patients in the early postoperative period to recall their health and well-being in the week before surgery, and to complete the baseline questionnaire based on this recall. However, the reliability of scores collected at these two-time points is uncertain. Therefore, this study was conducted to compare baseline QoL scores collected either preoperatively or postoperatively within a cohort of patients who underwent major cancer surgery by comparing the mean differences via large cohort of prospective exenteration patients whose baseline measurements are recorded situationally at the different time points.

## Main text

This study used data from a prospectively maintained clinical and QoL database [7, 8]. The database is maintained through REDCap, and is managed by the Surgical Outcomes Research Centre (SOuRCe). Patients’ clinical information and QoL data is collected at baseline (for this study either preoperatively or postoperatively) and then at 6, 12, 18, 24, 30, 36, 48 and 60 months postoperative. In brief, participants for this study included patients with advanced primary or recurrent rectal cancer who underwent pelvic exenteration at the Royal Prince Alfred Hospital (RPAH) Sydney, between 2008 and 2016. Ethics approval for the QoL study was granted by the Royal Prince Alfred Research Human Research Ethics Committee (Approval Number X16-0272).

### Inclusion and exclusion criteria

Patients eligible for the study was adults aged 18 years and over with non-metastatic curable locally advanced or locally recurrent cancer arising from the pelvis. Although the type of cancer is not restricted, what these cancers have in common is the need for radical multivisceral en bloc resection. Patients were also excluded if they had cognitive impairment such that they are unable to give informed consent or inadequate English to complete self-reported outcome measures.

Pelvic exenteration was defined as en bloc resection of at least three major pelvic structures which may comprise of a major pelvic organ (e.g. rectum, uterus, bladder etc.) and/or pelvic neurovascular structure, soft tissue or bony structure (e.g. iliac vessels, obturator internus, sciatic nerve roots, sacrum etc.).

### Patient characteristics and quality of life measurements

Data collection at study enrolment included patient demographics, relevant clinical information as well as QoL data. For logistical reasons described above, some patients did not complete the QoL measures prior to surgery. This group of patients then completed QoL measures in the early postoperative period. Patients were specifically instructed to answer the questionnaires based on their recollection of their preoperative QoL status. The QoL questionnaire used at preoperative or postoperative was identical (i.e. same instruments were used).

The SF-36 was used to evaluate health related QoL [9]. It is a broad measure compared with other patient-reported outcome measures, which are either disease-, treatment- or symptom-specific, and provides two summary scales (physical and mental component summary scales) plus eight domain-specific subscales (vitality; physical functioning; bodily pain; general health perceptions; physical role functioning; emotional role functioning; social role functioning; and mental health). QoL data was scored for the preoperative and postoperative groups using SF-36 Scoring Software. Higher SF-36 scores indicate better QoL.

### Statistical analysis

Baseline pre- and postoperative demographics, clinical characteristics and QoL scores were summarised as mean ± standard deviation for continuous outcomes or as frequencies (percentage) for dichotomous outcomes. Differences between the pre- and postoperative group scores were assessed using Chi squared tests (dichotomous outcomes) or T tests (continuous outcomes) with *P* < 0.05 considered statistically significant. All analyses were performed using SPSS version 22 (SPSS, inc., Chicago, IL).

## Results

From January 2008 to December 2016, 446 patients underwent pelvic exenteration at RPAH. Of these a total of 283 (63.5%) patients were eligible and recruited into the study. The baseline self-reported questionnaire was completed by 133 patients preoperatively and by 150 patients postoperatively (recalled as preoperative) during this study period. The demographic and clinical characteristics of the included patients are described in Table 1. The mean age was 59.5 (SD 12.13) years. Most demographic and clinical characteristics were comparable between the pre- and postoperative groups, except for country of birth (P = 0.027) and language spoken at home (P = 0.020) (Table 1).

**Table 1:** Participants demographic and clinical characteristics

Characteristics	Time point baseline questionnaire was completed		P value
	Preoperative (n = 133)	Postoperative (n = 150)	
Age (years), mean ± SD	60.6 ± 11.1	58.5 ± 12.9	0.144
Gender, male (%)	81 (60.9)	87 (58.0)	0.798
Country of birth, n (%)			0.027
Australia	105 (78.9)	103 (68.7)	
Overseas	28 (21.1)	46 (30.7)	
Missing data	–	1 (0.7)	
Language spoken at home, n (%)			0.020
English	130 (97.7)	142 (94.7)	
Other	3 (2.3)	8 (5.3)	
Marital status, n (%)			0.157
Single/divorced/widowed	37 (27.8)	45 (30.0)	
Married/living with partner	96 (72.2)	103 (68.7)	
Missing data	–	2 (1.3)	
Highest level of education, n (%)			0.230
Undergraduate	97 (72.9)	120 (80.0)	
Post graduate	32 (24.1)	27 (18.0)	
Missing data	4 (3.0)	3 (2.0)	
Employment status, n (%)			0.944
Employed	50 (37.6)	59 (39.3)	
Unemployed	82 (61.7)	89 (59.3)	
Missing data	1 (0.8)	2 (1.3)	
Health cover, n (%)			0.182
Private	70 (52.6)	72 (48.0)	
No private health	63 (47.4)	78 (52.0)	
Type of cancer, n (%)			0.054
Recurrent	93 (69.9)	80 (53.3)	
Advanced primary	40 (30.1)	69 (46.0)	
Missing data	–	1 (0.7)	

**Fig. 1:**
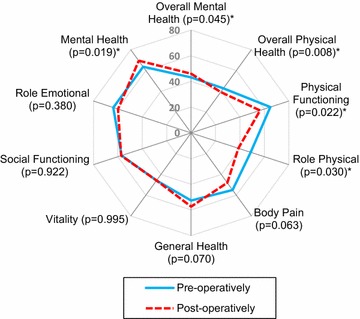
Comparison between preoperative and postoperative quality of life scores (*P < 0.05)

Comparison of baseline QoL scores collected pre- and postoperatively are summarised in Table 2 and Fig. 1. There were statistically significant differences between baselines QoL scores collected pre- and postoperatively on the physical health (mean ± SD preoperative = 42.79 ± 10.25 vs postoperative = 39.29 ± 11.3; P = 0.045) and mental health components (mean ± SD preoperative = 43.19 ± 11.67 vs postoperative = 46.00 ± 11.38; P = 0.008). On the QoL domain-specific subscales, differences were noted in physical functioning (mean ± SD preoperative = 64.94 ± 28.17 vs postoperative = 56.47 ± 32.74; P = 0.022), role physical (mean ± SD preoperative = 48.80 ± 34.78 vs postoperative = 38.68 ± 41.00; P = 0.030), and mental health domains (mean ± SD preoperative = 63.57 ± 20.45 vs postoperative = 69.36 ± 20.26; P = 0.019). The other domain-specific scores were similar.

**Table 2:** Preoperative and postoperative (perceived preoperative) quality of life scores

SF-36 domains	Preoperative group (N = 133)^a^	Postoperative group (N = 150)^a^	Effect size	
			MD (95% CI)	P value
Physical functioning	64.94 ± 28.17	56.47 ± 32.74	− 8.47 (− 15.66 to − 1.28)	0.022
Role physical	48.80 ± 34.78	38.68 ± 41.00	− 10.12 (− 19.08 to − 1.16)	0.030
Bodily pain	54.97 ± 29.98	47.93 ± 32.62	− 7.04 (− 14.40 to 0.32)	0.063
General health	52.64 ± 21.78	57.31 ± 21.12	4.67 (− 0.35 to 9.69)	0.070
Vitality	45.81 ± 22.96	45.82 ± 23.71	0.01 (− 5.47 to 5.49)	0.995
Social functioning	56.92 ± 32.09	57.30 ± 31.71	0.38 (− 7.10 to 7.86)	0.922
Role emotional	63.79 ± 32.38	59.86 ± 40.40	− 3.93 (− 12.57 to 4.71)	0.380
Mental health	63.57 ± 20.45	69.36 ± 20.26	5.79 (1.02 to 10.56)	0.019
Overall physical health	42.79 ± 10.25	39.29 ± 11.30	− 3.50 (− 6.04 to − 0.96)	0.008
Overall mental health	43.19 ± 11.67	46.00 ± 11.38	2.81 (0.11 to 5.51)	0.045

^a^ Scores are mean ± standard deviation (higher scores indicate better quality of life); *MD* mean difference (negative values favours preoperative group); *CI* confidence intervals

## Discussion

Collecting QoL data in the preoperative setting can be challenging. The purpose of this study was to determine whether QoL measures collected in the early postoperative period based on patients’ recall of their preoperative QoL status are comparable with scores obtained preoperatively. Unfortunately, this study found that QoL scores based on postoperative recall were lower for physical health but higher for mental health compared with scores obtained from patients preoperatively.

Postoperatively, the perception of QoL physical scores, including the overall physical health component, physical functioning and role physical based on recall were worse for the group assessed postoperatively than preoperatively. Conversely, QoL mental health scores, including the mental health component and mental health specific domains were perceived better postoperatively when compared to preoperatively. Other specific domains, such as bodily pain, general health, vitality, social functioning and role emotional were equally perceived by the patients, independently of the time point.

To our knowledge this is the first study to investigate whether the recall of preoperative QoL scores are different postoperatively in patients undergoing major cancer surgery. Interestingly, when the preoperative SF-36 scores are compared with postoperative (perceived preoperative) scores it reveals that the overall physical and mental health domains including the overall physical and mental health scores were perceived differently. This may suggest that cancer patients that undergo major surgery are mentally better postoperatively than preoperatively, due to the fact that they survived the fears of a major and complex surgery and can potentially now see a long-term survival. While on the other hand, in the postoperative group, the physical components were perceived worse than the preoperative group, this may be related to the extreme physical limitations post operation, including physical, bowel and sexual dysfunction, faecal and urinary incontinence. While they were emphatically asked to recall their preoperative state it is obvious the postoperative state has confounded the perception positively mentally and negatively physically. When compared with other studies investigating QoL following major cancer surgery, the preoperative overall scores of the physical (mean ± SD = 43.7 ± 10.3) and mental health (mean ± SD = 42.9 ± 11.6) components were similar to our preoperative group scores [7]. This may suggest that patients undergoing major surgery may perceive their preoperative status differently postoperatively. Therefore, our findings support the collection of baseline QoL data preoperatively where possible. Our findings are limited by the study design, differences in the characteristics of the samples (i.e. country of birth and language spoken at home), and lack of generalizability due to the very specific type of cancer and surgery studied, as such, caution should be taken when interpreting these results.

From the results of this explanatory study, it is clear that continued research and the application of response shift on major cancer surgical field and outcomes are needed. In brief, future studies should consider measuring changes in QoL by examining some of the following research designs described: (i) pre-test/post-test [10, 11]; (ii) then-test [12, 13]; (iii) structural equation modelling [14–16]; (iv) anchoring vignettes [12, 15, 17, 18]. Furthermore, future studies should focus on the clinical application of response shift measurement and how this may be incorporated into clinical practice.

## Conclusion

Patients undergoing major cancer surgery perceive their preoperative physical and mental health scores of the SF-36 QoL questionnaire differently pre- and postoperatively. Future studies, collecting data preoperatively and immediately after surgery are warranted to support our findings.

## Limitations

This study consisted of a small sample of participants undergoing a complex and rare cancer procedure and therefore may not be generalised. Participants were grouped according to the period they answer their baseline questionnaire (i.e. preoperatively versus postoperatively), potentially resulting in high risk of bias. Caution should be taken when interpreting these results.

## Abbreviations

QoL: quality of life
RPAH: Royal Prince Alfred Hospital
SF-36: Short Form 36
SD: standard deviation
